# Calcineurin-responsive zinc finger 1 (Crz1) contributes to stress tolerance and virulence in the pathogenic fungus *Trichosporon asahii*

**DOI:** 10.1128/iai.00759-25

**Published:** 2026-03-31

**Authors:** Yuta Shimizu, Yasuhiko Matsumoto, Takashi Sugita

**Affiliations:** 1 Department of Microbiology, Meiji Pharmaceutical University 34779, Kiyose, Tokyo, Japan; University of Pennsylvania Perelman School of Medicine, Philadelphia, Pennsylvania, USA

**Keywords:** *T. asahii*, virulence, stress tolerance, infection, Crz1

## Abstract

The pathogenic fungus *Trichosporon asahii* causes severe invasive fungal infections in immunocompromised patients with neutropenia. In *Cryptococcus neoformans*, calcineurin-responsive zinc finger 1 (Crz1) functions as a transcription factor downstream of the calcineurin signaling pathway and regulates the expression of genes involved in stress resistance and virulence. In *T. asahii*, Cna1 and Cnb1, which are key components of the calcineurin pathway, contribute to various stress responses and virulence. The role of Crz1 in stress tolerance and virulence in *T. asahii*, however, has remained unclear. Here, we demonstrate that a *crz1* gene-deficient *T. asahii* mutant exhibited increased sensitivity to cell wall and endoplasmic reticulum stress. The *crz1* gene-deficient mutant was sensitive to Congo red and tunicamycin but not to dithiothreitol or sodium dodecyl sulfate. Moreover, the virulence of the *crz1* gene-deficient mutant in the silkworm infection model was reduced. These phenotypes of the *crz1* gene-deficient mutant were restored by reintroducing the *crz1* gene, confirming the association between Crz1 and these phenotypes. The half-maximal lethal dose of the *cnb1* gene-deficient *T. asahii* mutant was higher than that of the *crz1* gene-deficient mutant. These results suggest that Crz1 mediates the stress responses and virulence of *T. asahii*. The involvement of Crz1 in the virulence of *T. asahii* is small, however, compared with that of the calcineurin.

## INTRODUCTION

*Trichosporon asahii* is a dimorphic basidiomycete that is widely distributed in nature and is commonly isolated from soil, a variety of animal hosts, and the human gastrointestinal tract (1–6). *T. asahii* is an opportunistic pathogen that can cause severe invasive fungal infections, particularly in immunocompromised hosts, such as patients with neutropenia (7–9). The mortality rate of invasive fungal infections caused by *T. asahii* is 70%–80% (10). Because echinocandin-class antifungal agents commonly used in the clinical treatment of fungal infections exhibit minimal antifungal activity against *T. asahii*, breakthrough infections may occur in patients undergoing treatment with micafungin (11, 12). Furthermore, clinical isolates of *T. asahii* resistant to antifungal agents, such as amphotericin B and azoles, have been isolated (13, 14). Therefore, revealing the molecular mechanisms of *T. asahii* infection may contribute to the development of effective prevention and treatment strategies (9).

Calcineurin-responsive zinc finger 1 (Crz1) is a transcription factor containing a zinc finger DNA-binding domain (15–19). In *Cryptococcus neoformans*, a pathogenic basidiomycetous fungus closely related to *T. asahii*, Crz1 contributes to fungal virulence by activating the calcineurin signaling pathway (17, 18, 20–22). Dephosphorylation of Crz1 by the calcineurin–calmodulin complex enables its translocation from the cytoplasm to the nucleus (22, 23). Once in the nucleus, Crz1 regulates the expression of genes required for growth at 37°C and for resistance to membrane and cell wall stress in *C. neoformans* (17, 18, 20–22). Calcineurin is involved in the virulence of *T. asahii*, as well as its resistance to high-temperature stress, membrane stress, cell wall stress, and endoplasmic reticulum (ER) stress (24). Whether Crz1 in *T. asahii* plays a role in stress resistance and virulence, however, has remained unknown.

In this study, we generated a *crz1* gene-deficient *T. asahii* mutant and examined the effects of the *crz1* gene on the stress response and virulence of *T. asahii*. The *crz1* gene-deficient mutant exhibited normal growth but showed increased sensitivity to tunicamycin and Congo red. Moreover, the virulence of the *crz1* gene-deficient mutant against silkworms was reduced compared with that of the parental strain. These findings suggest that Crz1 in *T. asahii* contributes to resistance against various stresses and plays a role in its virulence.

## MATERIALS AND METHODS

### Reagents

Nourseothricin was purchased from JENA BIOSCIENCE GMBH (Dortmund, Germany). Hygromycin B was obtained from Tokyo Chemical Industry Co., Ltd. (Tokyo, Japan). Cefotaxime sodium, D-glucose, agar, NaCl, sorbitol, and dithiothreitol (DTT) were purchased from FUJIFILM Wako Pure Chemical Corporation (Osaka, Japan). Congo red was purchased from Sigma-Aldrich Co., LLC (St. Louis, MO, USA). G418 was obtained from Enzo Life Science, Inc. (Farmingdale, NY, USA). Hipolypeptone was purchased from NIHON PHARMACEUTICAL Co., LTD (Tokyo, Japan). Tunicamycin (TM) was purchased from Cayman Chemical Company (Ann Arbor, MI, USA). Sodium dodecyl sulfate (SDS) was obtained from NIPPON GENE Co., LTD. (Tokyo, Japan).

### *T. asahii* strains

*T. asahii* MPU129 Δ*ku70* was used as the parental strain (25). The *crz1* gene-deficient mutant of *T. asahii* and the *crz1* gene-complemented strain in which the *crz1* gene was reintroduced into the *crz1* gene-deficient mutant were used. Strain information is provided in Table 1.

**TABLE 1:** Strains used in this study

*T. asahii* strains	Relevant genotype	Background	Reference
MPU129 *ku70*∆ (Parent strain)	*ku70::nptII*	MPU129	Matsumoto et al. (25)
Parent expressing Crz1-mCherry	*ku70::nptII, crz1::crz1-mCherry, hph*	MPU129 *ku70*∆	This study
*crz1*∆	*ku70::nptII, crz1::NAT1*	MPU129 *ku70*∆	This study
Crz1 (Revertant)	*ku70::nptII, NAT1::crz1, hph*	MPU129 *ku70*∆ *crz1*∆	This study

### *T. asahii* culture condition

*T. asahii* strains were cultured as described previously (25). *T. asahii* strains were grown on Sabouraud dextrose agar (SDA; 1% hipolypeptone, 4% dextrose, and 1.5% agar) containing G418 (100 µg/mL) and cefotaxime (100 µg/mL) at 27°C for 1–2 days.

### Estimation of Crz1 in *T. asahii*

The Crz1 amino acid sequence of *Cryptococcus neoformans* was obtained from the National Center for Biotechnology Information (NCBI) (https://www.ncbi.nlm.nih.gov/). BLASTp was used to search for a homolog of *C. neoformans* Crz1 in *T. asahii* (https://blast.ncbi.nlm.nih.gov/Blast.cgi). Hypothetical protein A1Q1_05631, having the highest homology to *C. neoformans* Crz1, was identified as Crz1 of *T. asahii*.

### Homology analysis of Crz1 between *T. asahii* and other fungi

The Crz1 amino acid sequences of *T. asahii*, *Candida albicans*, *Aspergillus fumigatus*, *Cryptococcus neoformans*, and *Saccharomyces cerevisiae* were obtained from the NCBI. Homology analysis with these Crz1 proteins was performed using BLASTp. A phylogenetic tree was constructed following the maximum likelihood method using MEGA11 Software (https://www.megasoftware.net/).

### Construction of a *T. asahii* strain expressing the Crz1-mCherry fusion protein

A *T. asahii* strain expressing a Crz1-mCherry fusion protein was constructed as described previously (24–27). Fragments of approximately 1,000–1,500 bp in size corresponding to the 5′ and 3′ untranslated regions (UTRs) of the *crz1* gene were cloned upstream and downstream, respectively, of the *hph* selectable marker in the pUC19 vector using the In-Fusion cloning method (In-Fusion HD Cloning Kit; Takara Bio Inc., Shiga, Japan). The 5′UTR(*crz1*)–*crz1-mCherry*–3′UTR(*crz1*) cassette was amplified by polymerase chain reaction (PCR) using the constructed plasmid. Competent *T. asahii* cells were prepared, as previously described (24–27). The PCR-amplified *crz1-mCherry* targeting fragment (2 µL) was introduced into 40 µL of competent parental *T. asahii* cells by electroporation using a Gene Pulser Xcell system (Bio-Rad Laboratories, Hercules, CA, USA) with a 2-mm gap cuvette under time-constant conditions (1,800 V, 5 ms), as described previously (24–27). After electroporation, cells were incubated in 500 µL of YPD medium supplemented with 0.6 M sorbitol at 27°C for 6 h, then plated onto SDA containing hygromycin B (200 µg/mL) and incubated at 27°C for 3–5 days. Hygromycin B–resistant colonies were isolated, genomic DNA was extracted, and correct integration of the *crz1-mCherry* fusion gene was confirmed by PCR. The primers used in this study are listed in Table 2.

**TABLE 2:** Primers used in this study

Primers	Nucleic acid sequence
pUC19-*crz1*(5′UTR)-*crz1*-*mCherry*-*hph*-*crz1*(3′UTR) (cloning)	
F Hygr (mCherry inf)	GGGCCCCCTGCGAGGATGTGAGC
R Crz1 (mCherry inf)	CACTGCAAGCGGCTTTTGCTCCTTGCCC
F mCherry (Crz1 inf)	CAAAAGCCGCTTGCAGTGATGGTCTCCAAGGGCGAGGAGGACAACATG
R mCherry (Hygr inf)	CATCCTCGCAGGGGGCCCTTACTTGTAGAGCTCGTCCATGCCGCCGG
Amplification of *crz1*-*mCherry* cassette for electroporation	
F Crz1 5UTR (pUC19 inf)	GAGCTGATACCGCTCGCCGCCTGGATCTCGGCACGCATCTCCTC
R Crz1 3UTR (pUC19 inf)	GTTTCGGTGATGACGGTGCCCCACCTTTCGCTCCAGCTTTCGC
Primers-1 for *crz1*-*mCherry* genotyping	
F Crz1 insert check	GCAGCAGCAGGGAGCTGGCAACTTTGGC
R Crz1 outside check	CCAGGTGGCTGAGCCAAGGCTGGAATGG
Primers-2 for *crz1-mCherry* genotyping	
F mCherry (crz1 inf)	ATGGTCTCCAAGGGCGAGGAGGACAACATG
R Crz1 outside check	CCAGGTGGCTGAGCCAAGGCTGGAATGG
Primers-3 for *crz1*-*mCherry* genotyping	
F Hygr (for cnb1 3UTR typing)	CAGGGTCGATGCGACGCAATCGTCCGATCC
R Crz1 outside check	CCAGGTGGCTGAGCCAAGGCTGGAATGG
Primers-4 for *crz1-mCherry* genotyping	
F crz1 insert check	GCAGCAGCAGGGAGCTGGCAACTTTGGC
R mCherry (outside typing)	GCCACCCTTGGTGACCTTGAGCTTGGCAG
pUC19-*crz1*(5′UTR)-*NAT1*-*crz1*(3′UTR) for cloning	
pUC19-*NAT1* (1st cloning)	
F pUC19	CACCGTCATCACCGAAACGCGCG
R pUC19	GGCGAGCGGTATCAGCTCACTCAAAGGC
F NAT1 (pUC19 inf)	GAGCTGATACCGCTCGCC GCTGCGAGGATGTGAGCTGGAGAGC
R NAT1(pUC19 inf)	GTTTCGGTGATGACGGTGGAAGAGATGTAGAAACTAGCTTCCTGGTTTCAGAG
pUC19-*crz1*(5′UTR)-*NAT1* (2nd cloning)	
F *NAT1*	GCTGCGAGGATGTGAGCTGGAGAGC
R pUC19 (long primer)	GGCGAGCGGTATCAGCTCACTCAAAGGCGGTAATACGGT
F Crz1 5UTR (pUC19 inf)	GAGCTGATACCGCTCGCCGCCTGGATCTCGGCACGCATCTCCTC
R Crz1 5UTR (NAT1 inf)	AGCTCACATCCTCGCAGCCGTTGTGCCGTTGTGCCAGGCGAG
pUC19-*crz1*(5′UTR)-*NAT1*-*crz1*(3′UTR) (3rd cloning)	
F pUC19	CACCGTCATCACCGAAACGCGCG
R NAT1	GAAGAGATGTAGAAACTAGCTTCCTGGTTTCAGAG
F Crz1 3UTR (NAT1 inf)	TAGTTTCTACATCTCTTCCCACCACTTGCACCGGTATGCTGGATGC
R Crz1 3UTR (pUC19 inf)	GTTTCGGTGATGACGGTGCCCCACCTTTCGCTCCAGCTTTCGC
Amplification of *crz1* cassette for electroporation	
F Crz1 5UTR (pUC19 inf)	GAGCTGATACCGCTCGCCGCCTGGATCTCGGCACGCATCTCCTC
R Crz1 3UTR (pUC19 inf)	GTTTCGGTGATGACGGTGCCCCACCTTTCGCTCCAGCTTTCGC
pUC19-*crz1*(5′UTR)-*crz1*-*hph*-*crz1*(3′UTR) for cloning	
pUC19-*crz1*(5′UTR)-*hph*-*crz1*(3′UTR) (1st cloning)	
F *crz1* 3UTR	CCACCACTTGCACCGGTATGCTGGATGC
R *crz1* 5UTR	CGTTGTGCCGTTGTGCCAGGCGAG
F Hygr (*crz1* 5UTR inf)	TGGCACAACGGCACAACGGGGCCCCCTGCGAGGATG
R Hygr (*crz1* 3UTR inf)	TACCGGTGCAAGTGGTGGGGATCCGAAGAGATGTAGAAAC
pUC19-*crz1*(5′UTR)-*crz1*-*hph-crz1*(3′UTR) (2nd cloning)	
F Hygr	GGGCCCCCTGCGAGGATG
R crz1 5UTR	CGTTGTGCCGTTGTGCCAGGCGAG
F Crz1 (5UTR inf)	TGGCACAACGGCACAACGGCCGTCACACTCACGATCCGATCGTCCACC
R Crz1 (Hygr inf)	CATCCTCGCAGGGGGCCCGTCTGGACCGAGACCCTCGCTCGCTACAG
Amplification of crz1 revertant cassette for electroporation	
F Crz1 5UTR (pUC19 inf)	GAGCTGATACCGCTCGCCGCCTGGATCTCGGCACGCATCTCCTC
R Crz1 3UTR (pUC19 inf)	GTTTCGGTGATGACGGTGCCCCACCTTTCGCTCCAGCTTTCGC
Primers-1 for *crz1* genotyping	
F Crz1 5UTR (pUC19 inf)	GTTTCGGTGATGACGGTGCCCCACCTTTCGCTCCAGCTTTCGC
R Crz1 3UTR (pUC19 inf)	GAGCTGATACCGCTCGCCGCCTGGATCTCGGCACGCATCTCCTC
Primers-2 for *crz1* genotyping	
F Crz1 insert check	GCAGCAGCAGGGAGCTGGCAACTTTGGC
R Crz1 insert check	GGTCCATCGTGCTGTACTGGTCCTCACCGC
Primers-3 for *crz1* genotyping	
F *NAT1* (for cnb1 3UTR typing)	GGACGGCGAGCAGGCGCTCTACATGAGC
R Crz1 outside check	CCAGGTGGCTGAGCCAAGGCTGGAATGG
Primers-4 for *crz1* genotyping	
F Hygr (for 3UTR typing)	CAGGGTCGATGCGACGCAATCGTCCGATCC
R Crz1 outside check	CCAGGTGGCTGAGCCAAGGCTGGAATGG

### Fluorescence microscopy of the Crz1-mCherry fusion protein

A *T. asahii* strain expressing the Crz1-mCherry fusion protein was cultured on SDA supplemented with cefotaxime (100 µg/mL) at 27°C for 2 days. *T. asahii* cells grown on SDA were suspended in physiologic saline (2 mL). The cell suspension (500 µL) was centrifuged at 10,000 rpm for 5 min. The pellets were resuspended in 300 µL of Sabouraud medium alone, Sabouraud medium supplemented with CaCl₂ (2 M), or Sabouraud medium supplemented with CaCl₂ (2 M) and tacrolimus (1 µg/mL). The cell suspensions were incubated at 40°C for 60 min. After incubation, 700 µL of 99.5% ethanol (FUJIFILM Wako Pure Chemical Corporation, Osaka, Japan) was added, and the samples were placed on ice for 2 h. Cells were then centrifuged at 14,000 rpm for 10 min, resuspended in 2 mL of phosphate-buffered saline, and passed through a 40-µm cell strainer. The filtrates were centrifuged again at 10,000 rpm for 5 min, and the pellets were resuspended in 500 µL of phosphate-buffered saline.

For nuclear staining, 20 µL of a 1:12,000 dilution of DAPI solution (DOJINDO Co., Ltd., Kumamoto, Japan) was mixed with 40 µL of the cell suspension and incubated in the dark for 30 min. Aliquots were mounted on glass slides, covered with coverslips, and observed using a fluorescence microscope (BZ-X800; Keyence Corporation, Osaka, Japan).

### Construction of the *crz1* gene-deficient *T. asahii* mutant and the complement strain

The *crz1* gene-deficient *T. asahii* mutant and complement strain were constructed according to previous reports (24–27). Approximately 1,000-1,500 bp of the 5′-UTR and 3′-UTR of the *crz1* gene were cloned upstream and downstream of the *NAT1* gene region of the pUC19-NAT1 vector by the In-Fusion method (In-Fusion HD Cloning Kit, Takara Bio Inc., Shiga, Japan). The *crz1* gene-targeting DNA fragment 5′-UTR (*crz1*)-NAT1-3’UTR (*crz1*) was amplified by PCR using the constructed plasmid. The *crz1* gene-targeting DNA fragment (2 µL) was introduced to competent cells (40 µL) of the *T. asahii* parent strain by electroporation. The electroporation condition used a time constant protocol (1,800 V, 5 ms) with a 2-mm gap cuvette (Bio-Rad Laboratories, Inc., CA, USA) and Gene Pulser Xcell (Bio-Rad Laboratories, Inc., CA, USA). The electroporated cells were then incubated in 500 µL of YPD medium containing 0.6 M sorbitol at 27°C for 3 h. After incubation, the cells were spread onto SDA containing nourseothricin (300 µg/mL) and cultured at 27°C for 3-5 days. Candidate colonies grown on the drug-containing SDA were isolated, and their genomes were extracted to confirm *crz1* gene deficiency by PCR.

To construct the *crz1* gene-complemented strain, the *crz1* gene and *hph* gene conferring hygromycin B resistance were cloned between the downstream region of the *crz1* 5′-UTR and the upstream region of the 3′-UTR in the targeting plasmid used for constructing the crz1 gene deletion mutant. The DNA fragment 5′-UTR (*crz1*)-*crz1-hph*-3′UTR (*crz1*) was amplified by PCR using the constructed plasmid. Competent cells (40 µL) of the *crz1* gene-deficient mutant were mixed with 2 µL of the PCR-amplified DNA fragment (5′-UTR (*crz1*)-*crz1-hph*-3′UTR (*crz1*)). To introduce the DNA into the cells, electroporation was performed using the time constant protocol (1,800 V, 5 ms). The cells were then suspended in 500 µL of YPD medium containing 0.6 M sorbitol and cultured at 27°C for 6 h. The cell suspension was spread onto YPD medium containing hygromycin B (200 µg/mL) and cultured at 27°C for 5 days. Colonies that grew were isolated, and their genomes were extracted to confirm reintegration of the *crz1* gene and *hph* gene by PCR. Primers used in this study are shown in Table 2.

### Stress sensitivity test

Stress sensitivity tests were performed according to the previous report (24, 26). *T. asahii* strains were cultured on SDA containing cefotaxime (100 µg/mL) at 27°C for 1 day. *T. asahii* cells were suspended in saline, and the cell suspension was adjusted to an absorbance of 1 at 630 nm. Ten-fold serial dilutions of each cell suspension were prepared using saline. For the temperature sensitivity test, cell suspension (5 µL) was spotted onto SDA. Photographs were taken after cultivation at 27°C, 37°C, and 40°C for 48 h. For the drug sensitivity test, cell suspension (5 µL) was spotted onto SDA containing SDS, Congo red, TM, and DTT. Photographs were taken after cultivation at 37°C for 48 h.

### Silkworm infection experiment

Silkworm infection experiments were performed as described previously (24, 26, 27). *T. asahii* strains were grown on YPD agar containing cefotaxime (100 µg/mL) at 27°C for 1 day. *T. asahii* cells were suspended in saline and filtered through a cell strainer with a 40-µm pore size. The absorbance of the pass-through *T. asahii* cells at 630 nm was adjusted to 0.4. The *T. asahii* cell suspension was diluted 400-fold with saline, and 50 µL of the diluted suspension was injected into the silkworm hemolymph from the dorsal side using a 1-mL tuberculin syringe (Terumo Medical Corporation, Tokyo, Japan). The infected silkworms were fed an artificial diet (Silkmate 2S) and maintained at 37°C with monitoring for 4 days.

The half-maximal lethal dose (LD_50_) of *T. asahii*, the dose required to kill half of the silkworms, was determined by modifying a previously reported method (26). *T. asahii* (1.6 × 10^1^ to 1.5 × 10^7^ cells/50 µL) was injected into the silkworm hemolymph, and the silkworms were reared at 37°C with Silkmate 2S. The number of surviving silkworms (*n* = 4 per group) was measured 72 h post-injection. The LD_50_ was determined using a simple logistic regression model based on curves obtained from three independent experiments analyzed with Prism software (GraphPad Software, LLC, San Diego, CA, USA, https://www.graphpad.com/scientific-software/prism/).

### Statistical analysis

All experiments were performed at least three times, and representative results are shown. The statistical significance of differences in the growth of *T. asahii* between groups was calculated by Student’s *t*-test. The significance of differences between groups in the silkworm infection experiment was calculated by the log-rank test based on graphs constructed using the Kaplan-Meier method with JMP Pro 17 (https://www.jmp.com/ja_jp/support/jmp-software-updates.html). *P* < 0.05 was considered statistically significant.

## RESULTS

### Identification of Crz1 protein in *T. asahii* and its conservation among fungi

**Fig 1:**
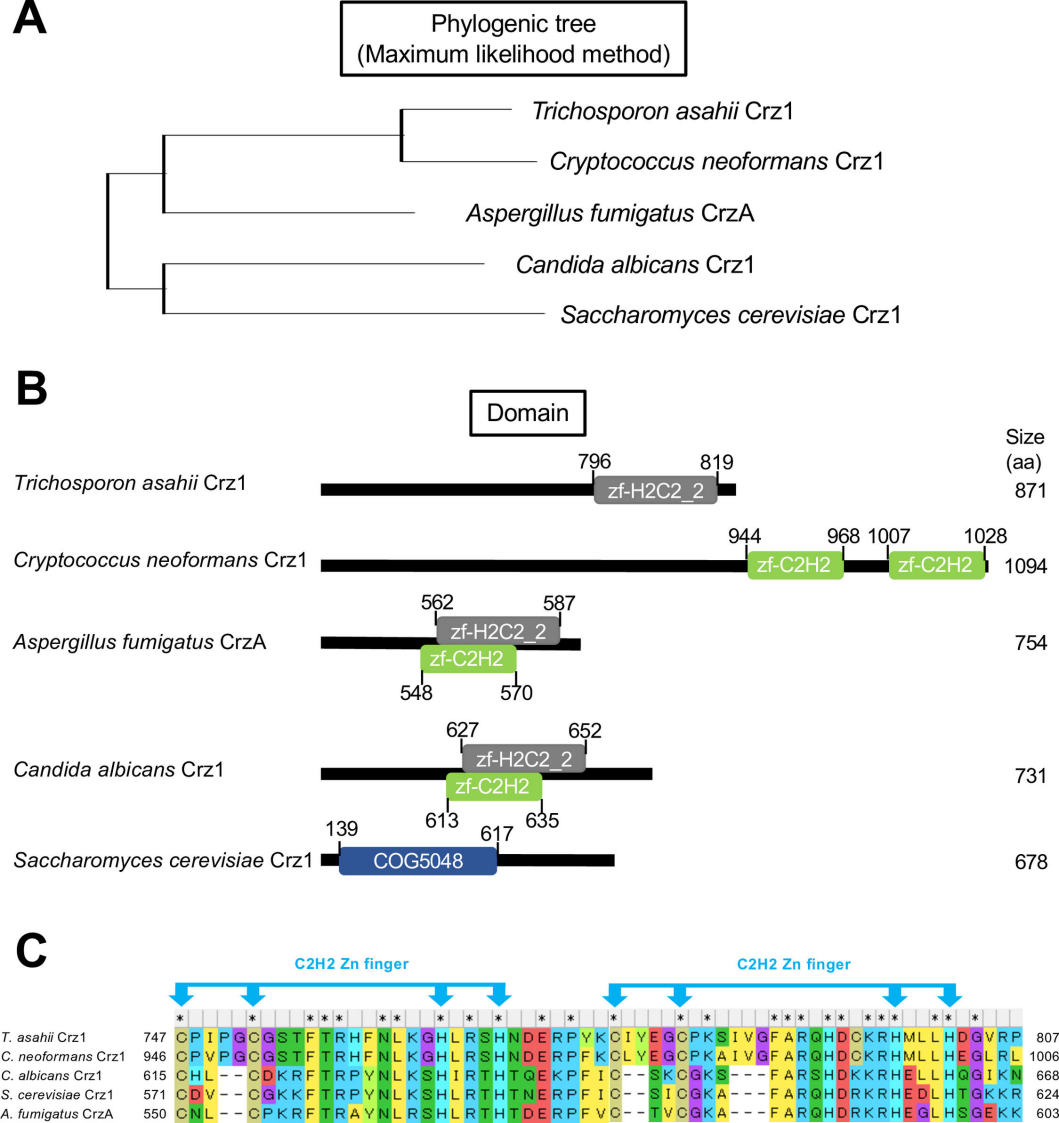
Homology analysis of Crz1 in *T. asahii* among various fungi. (**A**) Phylogenetic trees were constructed using the maximum likelihood method for Crz1 proteins of *T. asahii*, *C. neoformans*, *C. albicans*, *S. cerevisiae*, and CrzA of *A. fumigatus*. (**B**) Schematic representation of the domains present in Crz1 and CrzA proteins. zf-H2C2_2, zf-C2H2, and COG5048 are zinc finger domains. (**C**) Amino acid sequences of the zinc finger domain in Crz1 and CrzA proteins. The zinc finger domain contains two C2H2 Zn fingers. Amino acid alignment analysis and domain prediction were performed using MEGA11 Software.

We estimated *T. asahii* Crz1 based on *in silico* homology analysis. In *C. neoformans*, Crz1 is required for tolerance to various stressors as well as for virulence (18). A hypothetical protein of *T. asahii* (A1Q1_05631) was estimated as *T. asahii* Crz1. The percent identity and query cover of *T. asahii* A1Q1_05631 to the Crz1 protein of *C. neoformans* were 48.8% and 86%, respectively (Table 3). Therefore, we estimated that hypothetical protein A1Q1_05631 was *T. asahii* Crz1. Homology analysis of the amino acid sequence of the *T. asahii* Crz1 protein was conducted based on the genomic information of representative model fungi (*Saccharomyces cerevisiae*) and pathogenic fungi (*Cryptococcus neoformans*, *Candida albicans*, and *Aspergillus fumigatus*). The *T. asahii* Crz1 protein exhibited amino acid sequence identities of 49%, 48.8%, 42.1%, and 46% with the Crz1 proteins of *S. cerevisiae*, *C. neoformans*, *C. albicans*, and *A. fumigatus*, respectively (Table 3). A phylogenetic tree constructed from the amino acid sequence data of these proteins revealed that, among these fungi, the *T. asahii* Crz1 protein was most similar to the *C. neoformans* Crz1 protein (Fig. 1A). The *T. asahii* Crz1 protein contained the functional zinc finger domain similar to other fungal Crz1 proteins (Fig. 1B). Furthermore, two cysteines and two histidines essential for activity within the zinc finger domain were conserved across all fungi examined (Fig. 1C).

**TABLE 3:** Conservation of Crz1 among fungi including *T. asahii*

Species	Percent identity (%)	Query coverage (%)
*T. asahii*	100	100
*C. neoformans*	48.82	86
*A. fumigatus*	46.08	10
*S. cerevisiae*	49.07	11
*C. albicans*	42.11	13

### Calcineurin-dependent nuclear localization of the Crz1-mCherry fusion protein in *T. asahii*

**Fig 2:**
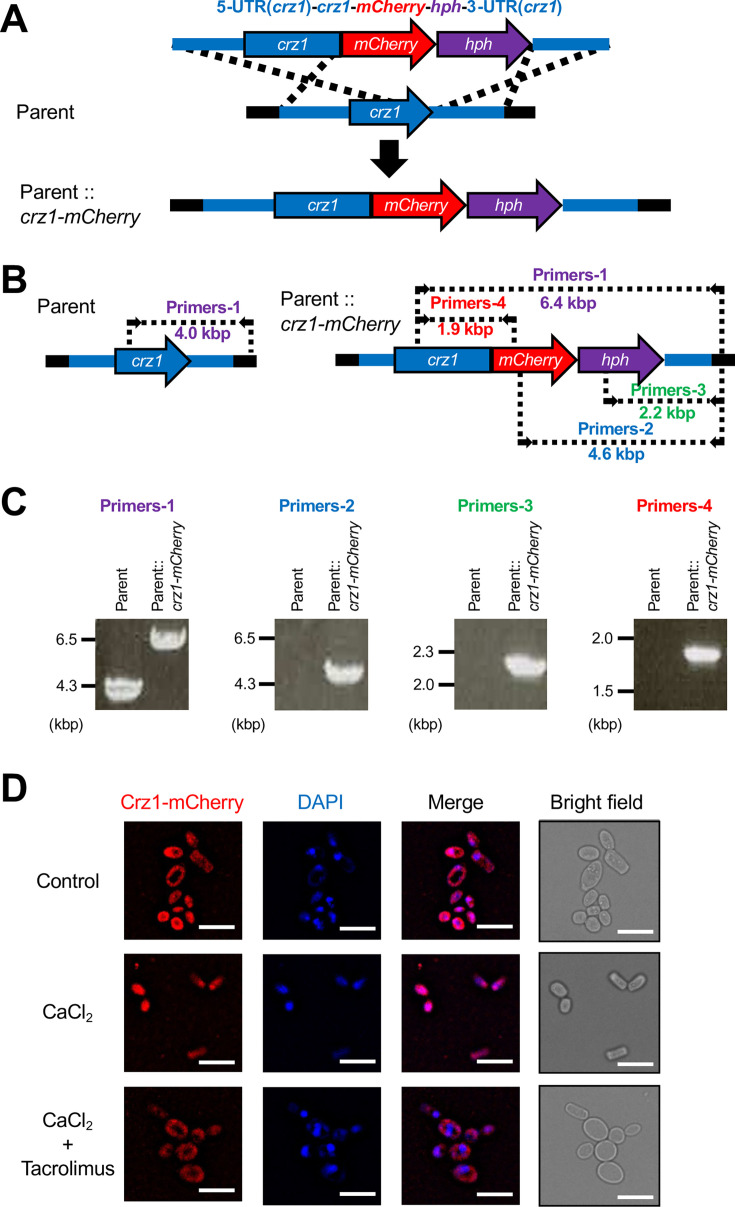
Inhibition of nuclear localization of the Crz1-mCherry fusion protein by tacrolimus. (**A**) Schematic representation of the strategy used to generate the *T. asahii* strain expressing the Crz1-mCherry fusion protein and the resulting genomic structure. (**B**) Positions of primers used for PCR verification of the *crz1-mCherry* fusion gene in the genome and expected sizes of the amplified DNA fragments. (**C**) Agarose gel electrophoresis of PCR products confirming the genotype of the *T. asahii* strain expressing the Crz1-mCherry fusion protein. (**D**) The Crz1-mCherry–expressing *T. asahii* strain was incubated with or without CaCl₂ (2 M) and tacrolimus (1 µg/mL) at 40°C for 1 h. Cells were fixed with 70% ethanol for 2 h, washed, and observed by fluorescence microscopy. Scale bar, 10 µm.

In several pathogenic fungi, nuclear localization of Crz1 is inhibited by tacrolimus, a calcineurin pathway inhibitor (16, 18, 28). We therefore investigated whether tacrolimus inhibits nuclear localization of Crz1 in *T. asahii*. We generated a *T. asahii* strain in which the endogenous *crz1* gene was replaced with a *crz1-mCherry* fusion gene by homologous recombination (Parent::*crz1-mCherry*) (Fig. 2A through C). The DNA fragment contained the *hph* gene, which confers resistance to hygromycin B, thereby allowing selection of transformants following genomic integration (Fig. 2A). Genomic DNA was extracted from hygromycin B-resistant transformants, and integration of the *crz1-mCherry* fusion gene was confirmed by PCR (Fig. 2B and C). Under conditions of CaCl₂ supplementation at 40°C, the Crz1-mCherry fusion protein localized predominantly to the nucleus (Fig. 2D). Moreover, treatment with tacrolimus inhibited nuclear localization of the Crz1-mCherry fusion protein (Fig. 2D). These results suggest that nuclear localization of the Crz1-mCherry fusion protein in *T. asahii* is mediated by calcineurin signaling.

### Construction of *crz1* gene-deficient *T. asahii* mutant

**Fig 3:**
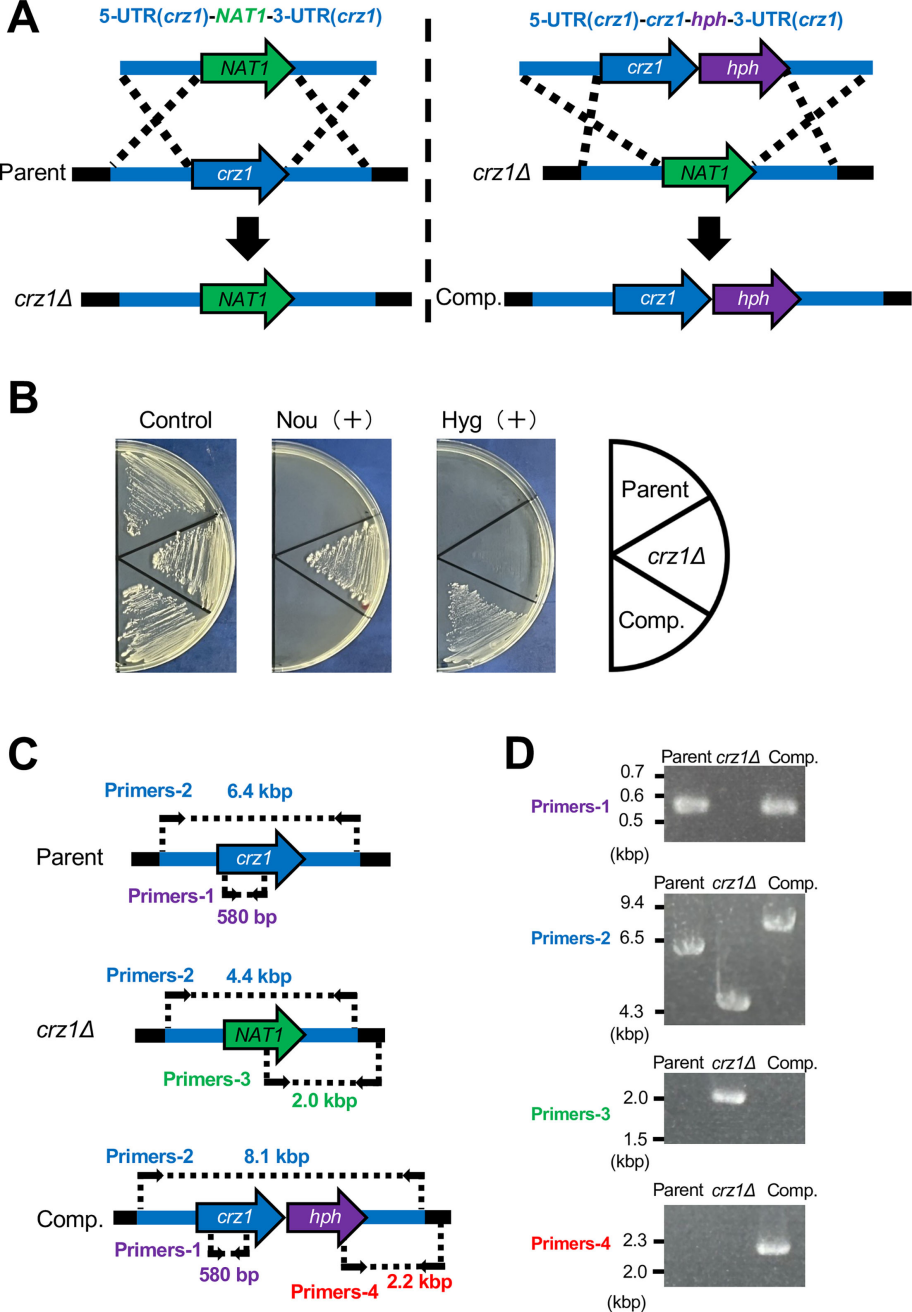
Establishment of *crz1* gene-deficient mutant and *crz1* gene-complemented *T. asahii* strains. (**A**) Schematic representation of the construction process for the *crz1* gene-deficient and the *crz1* gene-complemented mutant *T. asahii* strains. The genomic structures of the gene-deficient and complemented strains are also illustrated. (**B**) The parental strain (Parent), *crz1* gene-deficient strain (*crz1*Δ), and *crz1* gene-complemented strain (Comp.) were plated on SA medium containing nourseothricin (Nou; 100 µg/mL) or hygromycin B (Hyg B; 100 µg/mL) and incubated at 27°C for 2 days. (**C**) The positions of the primers and expected DNA fragment sizes are indicated to confirm the genomic structures of the *crz1* gene-deficient mutant. and complemented strain by PCR using extracted genomic DNA. (**D**) DNA electrophoresis of PCR products was performed to confirm the genotypes of the *crz1* gene-deficient mutant (*crz1*Δ) and its complemented strain (Comp.).

Next, we attempted to construct a *crz1* gene-deficient mutant of *T. asahii*. The DNA fragment required for *crz1* gene deficiency contained the *NAT1* gene, which confers resistance to nourseothricin (Fig. 3A). Insertion of this DNA fragment into the genome by homologous recombination confers resistance to nourseothricin (Fig. 3A). The DNA fragment was introduced into the *T. asahii* cells by electroporation, and transformants resistant to nourseothricin were obtained (Fig. 3B). Genomic DNA was extracted from the nourseothricin-resistant transformants, and *crz1* gene deficiency was confirmed by PCR (Fig. 3C and D). Using the same DNA recombination method, we generated the *crz1* gene-complemented strain from the *crz1* gene-deficient mutant (Fig. 3A through D). These results suggest that the *crz1* gene-deficient mutant and the *crz1* gene-complemented strain of *T. asahii* were successfully generated.

### Role of the *crz1* gene under high-temperature stress

**Fig 4:**
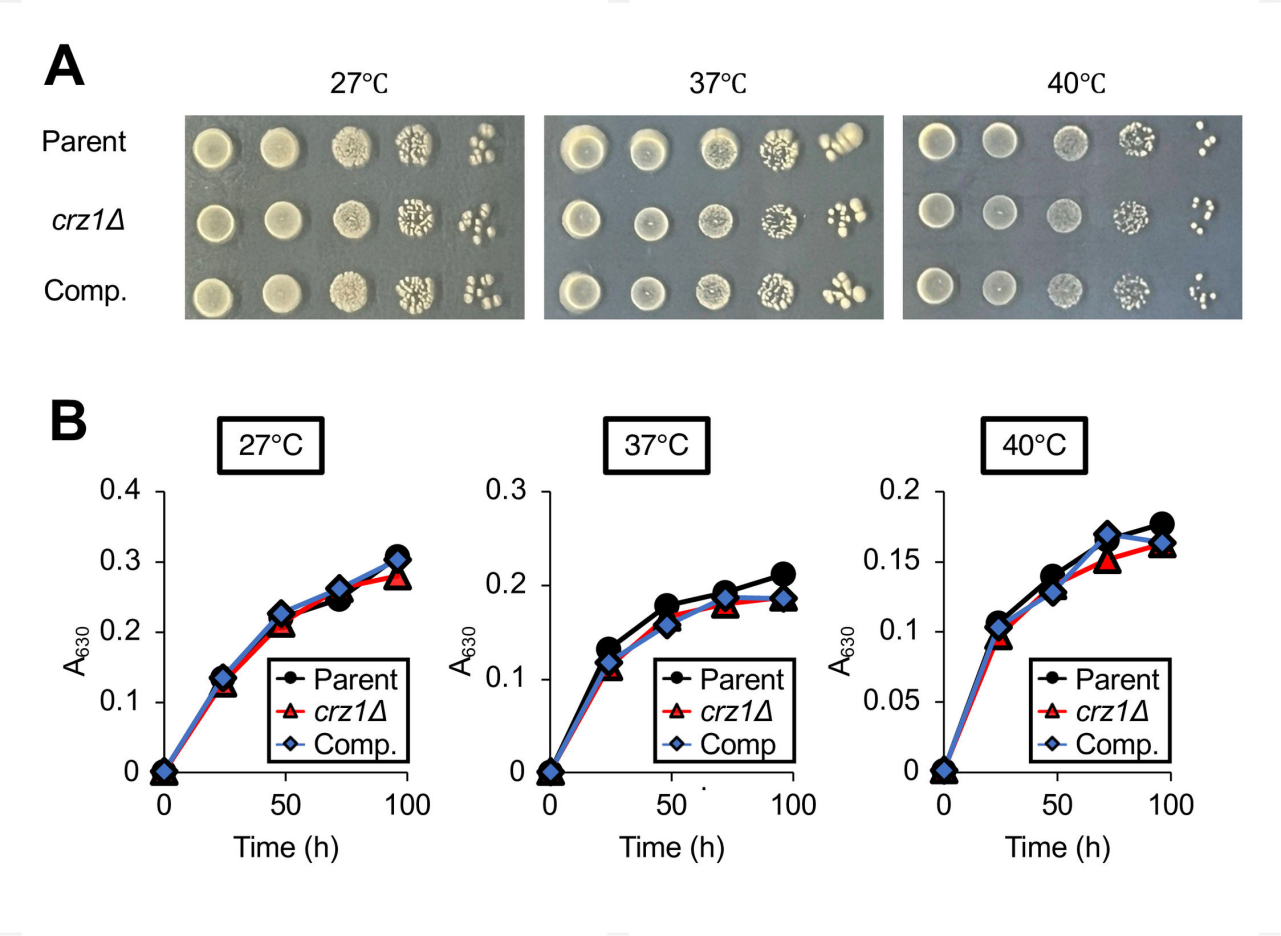
Temperature sensitivity of the *crz1* gene-deficient *T. asahii* mutant. (**A**) The parental (Parent), *crz1* gene-deficient mutant (*crz1*Δ), and *crz1* gene-complemented (Comp.) *T. asahii* strains were cultured on SA medium at 27°C for 2 days. *T. asahii* cells were suspended in saline to prepare a 10-fold serial dilution series. Five microliters of each cell suspension were spotted onto SA medium and incubated at 27°C, 37°C, or 40°C for 48 h. (**B**) The parental (Parent), *crz1* gene-deficient mutant (*crz1*Δ), and *crz1* gene-complemented (Comp.) *T. asahii* strains were cultured in Sabouraud dextrose liquid medium at 27°C, 37°C, or 40°C, and the optical density at 630 nm was measured.

The *crz1* gene-deficient strain of *C. neoformans* exhibits delayed growth at 39°C (18). Calcineurin is involved in the growth of *T. asahii* at 40°C (24). We investigated whether the *crz1* gene contributes to high-temperature stress response in *T. asahii*. The *crz1* gene-deficient mutant of *T. asahii* did not exhibit delayed growth at 40°C compared with the parental strain (Fig. 4). This finding suggests that the *crz1* gene is not involved in the tolerance of *T. asahii* to high-temperature stress.

### Role of the *crz1* gene in tolerance to various stresses

**Fig 5:**
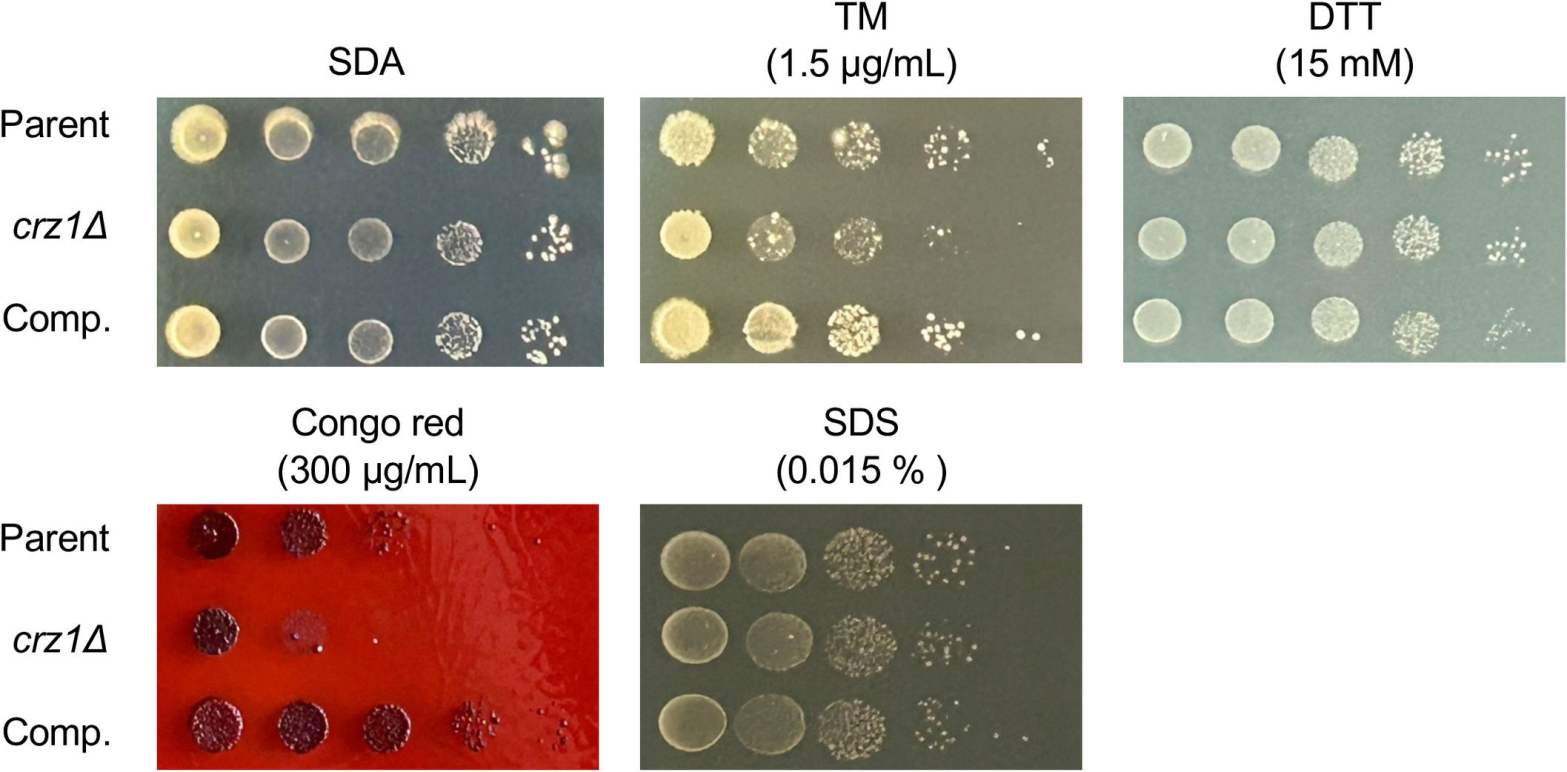
Stress sensitivity of the *crz1* gene-deficient *T. asahii* mutant. The parental (Parent), *crz1* gene-deficient mutant (*crz1*Δ), and *crz1* gene-complemented (Comp.) *T. asahii* strains were cultured on SA medium for 2 days. *T. asahii* cells were suspended in saline to prepare a 10-fold serial dilution series. The cell suspensions were spotted onto SDA medium containing either tunicamycin (TM) (1.5 µg/mL), dithiothreitol (DTT) (15 mM), Congo red (300 µg/mL), or sodium dodecyl sulfate (SDS) (0.015%) and incubated at 37°C for 48 h.

The *crz1* gene-deficient mutant of *C. neoformans* exhibits sensitivity to Congo red and SDS (24). Similarly, the *crz1* gene-deficient mutant of *C. albicans* shows sensitivity to SDS (28). Moreover, the *cnb1* gene-deficient mutant of *T. asahii* is sensitive to Congo red, SDS, TM, and DTT (24). We examined whether the *crz1* gene contributes to the response to various stressors. The *crz1* gene-deficient mutant of *T. asahii* exhibited delayed growth in the presence of Congo red, which induces cell wall stress, and TM, which induces ER stress, but not in the presence of SDS or DTT (Fig. 5). The *crz1* gene-complemented strain did not exhibit the phenotypes of cell wall stress and ER stress sensitivity observed in the *crz1* gene-deficient mutant (Fig. 5). Together, these findings suggest that the *T. asahii crz1* gene contributes to tolerance against cell wall stress and ER stress.

### Decreased virulence of the *crz1* gene-deficient mutant in a silkworm infection model

**Fig 6:**
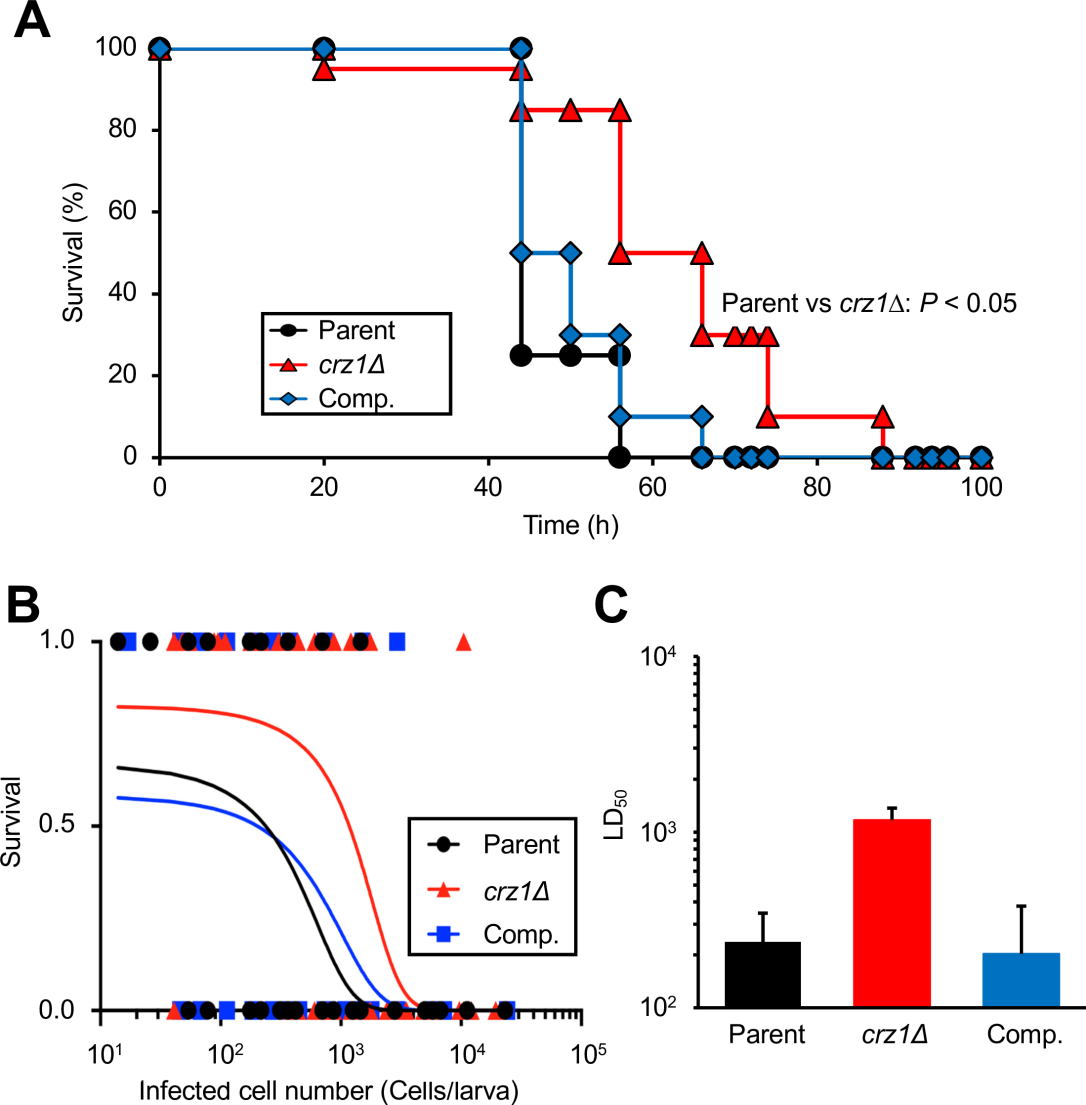
Virulence of the *crz1* gene-deficient *T. asahii* mutant against silkworms. (**A**) The parental (Parent; 160 cells/larva), *crz1* gene-deficient mutant (*crz1*Δ; 155 cells/larva), and *crz1* gene-complemented (Comp.; 210 cells/larva) *T. asahii* strains were injected into the hemocoel of silkworms, and their survival was monitored at 37°C for 96 h. The statistical significance of differences between the parental strain group and the *crz1* gene-deficient mutant group was determined by the log-rank test based on Kaplan-Meier survival curves. *P*-values < 0.05 were considered statistically significant. A total of 20 silkworms were used per group. (**B**) The survival rates of silkworms at 37°C were determined 72 h after *T. asahii* injection. Surviving and dead silkworms were scored as 1 and 0, respectively. A total of 48–64 silkworms were used per group. Curves were drawn by combining the results of three independent experiments using a simple logistic regression model. (**C**) The LD_50_ values were determined from the curves shown in panel **B**.

Crz1 in *C. neoformans*, *C. albicans*, and *A. fumigatus* is involved in its pathogenicity in mice (15, 18, 29). Therefore, we used a silkworm infection model to investigate whether the *crz1* gene deficiency affects the virulence of *T. asahii*. Silkworms injected with the *crz1* gene-deficient mutant survived longer than those injected with the parental strain (Fig. 6A). The LD_50_ value of the *crz1* gene-deficient mutant was approximately five times higher than that of the parental strain (Fig. 6B and C). This phenotype was not observed in the complemented strain (Fig. 6). These findings suggest that the *crz1* gene contributes to the virulence of *T. asahii* against silkworms.

### Comparison of phenotypes between *crz1* and *cnb1* gene-deficient *T. asahii* mutants

**Fig 7:**
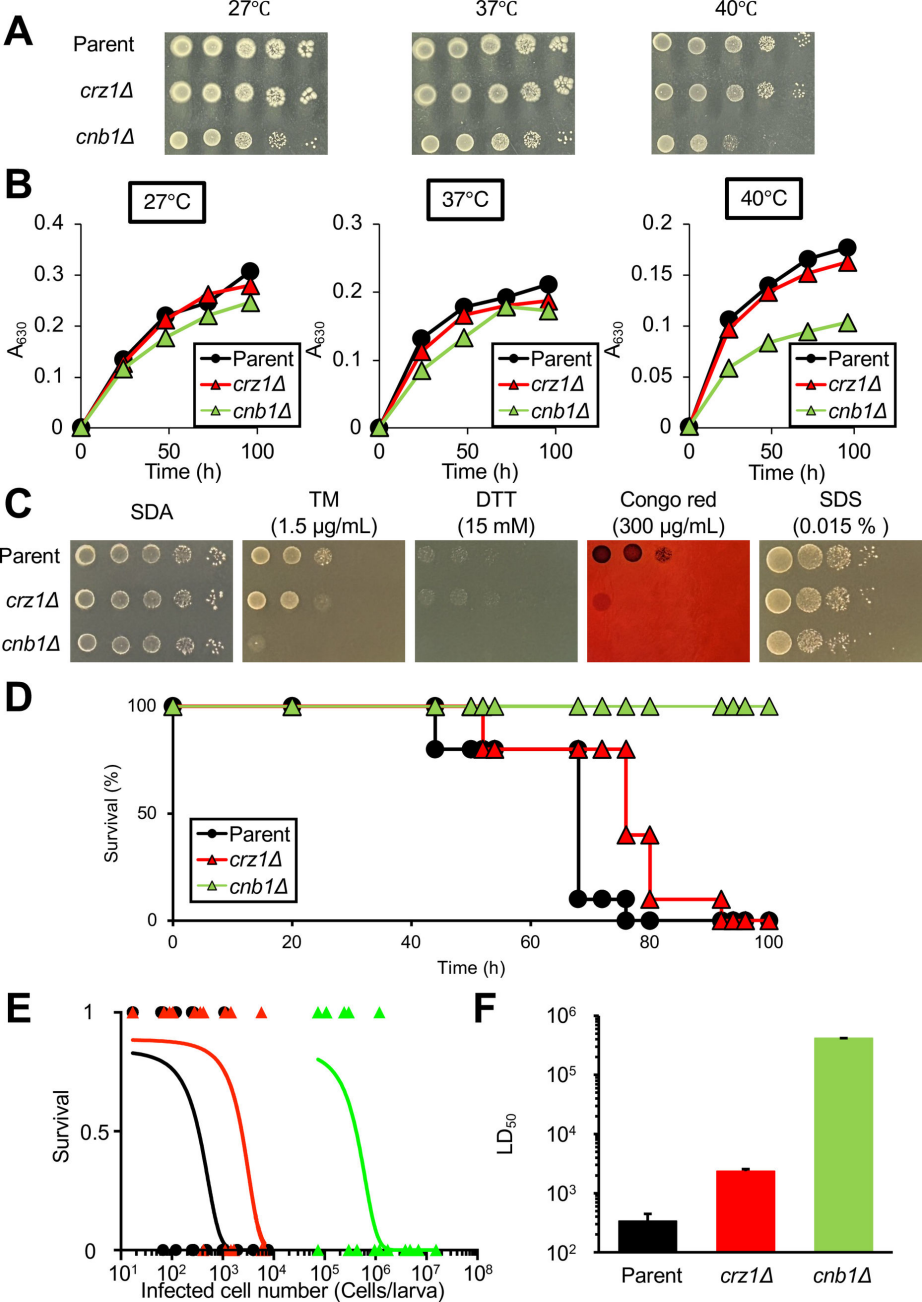
Comparison of the *crz1-* and *cnb1*-gene-deficient *T. asahii* mutant phenotypes. (**A**) The parental (Parent), *crz1* gene-deficient mutant (*crz1*Δ), and *cnb1* gene-deficient mutant (*cnb1*Δ) *T. asahii* strains were cultured on SDA at 27°C for 2 days. *T. asahii* cells were suspended in saline to prepare a 10-fold serial dilution series. Five microliters of each cell suspension were spotted onto SA medium and incubated at 27°C, 37°C, or 40°C for 48 h. (**B**) The parental (Parent), *crz1* gene-deficient mutant (*crz1*Δ), and *cnb1* gene-deficient mutant (*cnb1*Δ) *T. asahii* strains were cultured in Sabouraud dextrose liquid medium at 27°C, 37°C, or 40°C, and the optical density at 630 nm was measured. (**C**) The parental (Parent), *crz1* gene-deficient mutant (*crz1*Δ), and *cnb1* gene-deficient mutant (*cnb1Δ*) *T. asahii* strains were cultured on SDA for 2 days. *T. asahii* cells were suspended in saline to prepare a 10-fold serial dilution series. The cell suspensions were spotted onto SDA containing either tunicamycin (TM) (1.5 µg/mL), dithiothreitol (DTT) (15 mM), Congo red (300 µg/mL), or sodium dodecyl sulfate (SDS) (0.015%) and incubated at 37°C for 72 h. (**D**) The parental (Parent; 278 cells/larva), *crz1* gene-deficient mutant (*crz1*Δ; 298 cells/larva), and *cnb1* gene-deficient mutant (*cnb1*Δ; 400 cells/larva) *T. asahii* strains were injected into the silkworm hemolymph, and their survival was monitored at 37°C for 96 h. The statistical significance of differences between the parental strain group and the *crz1* gene-deficient mutant group was determined by the log-rank test based on Kaplan-Meier survival curves. *P*-values < 0.05 were considered statistically significant. Each group contained 20 silkworms. (**E**) Survival rates of silkworms at 37°C were determined 72 h after *T. asahii* injection. Surviving and dead silkworms were scored as 1 and 0, respectively. Each group contained 48–64 silkworms. Curves were drawn by combining the results of three independent experiments using a simple logistic regression model. (**F**) The LD_50_ values were determined from the curves shown in panel **E**.

In *C. neoformans*, Crz1 functions as a downstream factor of calcineurin (18). Next, we compared the phenotypes between *crz1* and *cnb1* gene-deficient *T. asahii* mutants. The *cnb1* gene-deficient mutant exhibited delayed growth at 40°C, whereas the *crz1* gene-deficient mutant did not show sensitivity at 40°C (Fig. 7A and B). The *cnb1* gene-deficient mutant was sensitive to TM, dithiothreitol (DTT), Congo red, and SDS. The sensitivity of the *crz1* gene-deficient mutant to TM and Congo red was lower than that of the *cnb1* gene-deficient mutant (Fig. 7C). While the *crz1* gene-deficient mutant exhibited reduced virulence in silkworms, the LD_50_ value of the *crz1* gene-deficient mutant in the silkworm infection system was 100-fold lower than that of the *cnb1* gene-deficient mutant (Fig. 7D through F). These results suggest that the phenotypes of the *crz1* gene-deficient *T. asahii* mutant were weaker than those of the *cnb1* gene-deficient *T. asahii* mutant.

## DISCUSSION

In this study, we generated a *T. asahii crz1* gene-deficient mutant and demonstrated that the mutant exhibited reduced tolerance to several stressors and reduced virulence in the silkworm infection model. These findings suggest that both virulence and stress resistance are regulated through Crz1-dependent pathways in *T. asahii*.

A hypothetical protein, A1Q1_05631, in *T. asahii* was designated as Crz1. Using the *C. neoformans* Crz1 amino acid sequence as a query, BLAST analysis identified A1Q1_05631 as the most homologous protein in *T. asahii* (Fig. S1). The query coverage of A1Q1_05631 was 75%, whereas that of all other candidate proteins was less than 15% (Fig. S1). Moreover, A1Q1_05631 exhibited a markedly lower E-value (e⁻¹⁶¹) compared with those of other proteins, which were all greater than e⁻¹³ (Fig. S1). Furthermore, a BLAST search using the A1Q1_05631 amino acid sequence identified Crz1 or CrzA homologs in other pathogenic fungi (Fig. S2). Based on these sequence similarity analyses, we estimated A1Q1_05631 as Crz1 in *T. asahii*. In *C. neoformans*, deletion of the region encoding residues 451–456 (PMICIQ), corresponding to the PxIxIT motif, abolishes nuclear translocation of Crz1 (18). Therefore, A1Q1_05631 in *T. asahii* contains a conserved PxIxIT motif (PMITIQ) at residues 336–341 (Fig. S3), suggesting that this protein is capable of nuclear translocation. Furthermore, we demonstrated that nuclear localization of the Crz1-mCherry fusion protein in *T. asahii* is mediated by calcineurin signaling. These sequence-based and functional analyses support the designation of *T. asahii* A1Q1_05631 as Crz1.

The *T. asahii crz1* gene contributes to its stress responses against TM and Congo red but not against high temperature, DTT, or SDS. TM induces ER stress (30), and Congo red induces cell wall stress (31). Therefore, Crz1 might regulate ER and cell wall stress tolerance in *T. asahii*. In *C. neoformans*, Crz1 has crucial roles in its tolerance to high temperature, TM, Congo red, DTT, and SDS (17, 18, 20, 32, 33). Moreover, *crz1* gene deficiency leads to increased sensitivity to TM and SDS in *C. albicans* (16, 28, 34). Therefore, Crz1 proteins in *T. asahii*, *C. neoformans*, and *C. albicans* commonly contribute to the response to ER stress induced by TM. On the other hand, the *crz1* gene is required for SDS tolerance in *C. neoformans* and *C. albicans* but not in *T. asahii*. In *C. neoformans,* but not *T. asahii,* high temperature sensitivity is increased by *crz1* gene deficiency. The phenotypic differences observed among pathogenic fungi might be due to species-specific regulatory mechanisms involving *crz1*. Further studies are needed to compare how Crz1 regulates gene expression across different fungal species. Moreover, the observed phenotypes were confirmed using three independent *T. asahii crz1* gene-deficient mutants (Fig. S4). None of the three independent *crz1* deletion mutants exhibited increased sensitivity to antifungal agents tested in this study (Table S1). Together, these results suggest that Crz1 is not required for antifungal drug tolerance in *T. asahii*.

The *crz1* gene contributes to the virulence of *T. asahii* against silkworms. In *C. neoformans*, *C. albicans*, and *A. fumigatus*, Crz1 is required for their virulence in mouse infection experiments (15, 18, 29). Therefore, Crz1 regulates the expression of genes required for virulence in various pathogenic fungi, including *T. asahii*. The calcineurin and Hog1 signaling pathways have important roles in *T. asahii* virulence (24, 26). Therefore, understanding the relationships between Crz1 and other signaling pathways in *T. asahii* virulence is an important goal.

The phenotypes of the *crz1* gene-deficient mutant were weaker than those of the *cnb1* gene-deficient mutant, which is a calcineurin-deficient mutant. Crz1 is regulated by calcineurin through dephosphorylation (23). Therefore, the phenotypes of the *crz1* gene-deficient mutant and the *cnb1* gene-deficient mutant were compared. The *cnb1* gene-deficient *T. asahii* mutant exhibited sensitivity to high temperature, TM, DTT, Congo red, and SDS (21, 22). Growth of the *cnb1* gene-deficient mutant was delayed under exposure to high temperature, TM, DTT, Congo red, or SDS, in comparison with the *crz1* gene-deficient mutant. Moreover, the LD_50_ value of the *cnb1* gene-deficient mutant was more than 100-fold higher than that of the *crz1* gene-deficient mutant. Therefore, the contribution of Crz1 to tolerance to various stressors and to the virulence of *T. asahii* was small compared with that of calcineurin. We hypothesize that calcineurin regulates *T. asahii* virulence via Crz1-dependent and independent mechanisms. Further investigation into the roles of calcineurin and Crz1 in *T. asahii* virulence will be an important focus of future research.

In conclusion, Crz1 mediates tolerance to various stressors as well as virulence in *T. asahii*, but its contribution is smaller than that of calcineurin. Therefore, transcription factors other than Crz1 may act downstream of calcineurin.

## Data Availability

Data will be made available upon reasonable request.

## SUPPLEMENTAL MATERIAL

Data set S1 iai.00759-25-s0001.xlsx For Fig. 4, 6, and 7.

Supplemental material iai.00759-25-s0002.pdf Table S1; Fig. S1 to S4.

ASM does not own the copyrights to Supplemental Material that may be linked to, or accessed through, an article. The authors have granted ASM a non-exclusive, world-wide license to publish the Supplemental Material files. Please contact the corresponding author directly for reuse.
