# Supplementary material for: Calcineurin-responsive zinc finger 1 (Crz1) contributes to stress tolerance and virulence in the pathogenic fungus Trichosporon asahii
Source: Infect Immun. 2026 Mar 31;94(5):e00759-25. doi: 10.1128/iai.00759-25 (PMC13163193; doi:10.1128/iai.00759-25)
Supplement: Supplemental material — Table S1; Fig. S1 to S4. [file iai.00759-25-s0002.pdf]

# Calcineurin-responsive zinc finger 1 (Crz1) contributes to stress tolerance and virulence in the pathogenic fungus *Trichosporon asahii*

Yuta Shimizu[1], Yasuhiko Matsumoto[1]*, Takashi Sugita[1]

[1]Department of Microbiology, Meiji Pharmaceutical University, 2-522-1, Noshio, Kiyose, Tokyo 204-8588, Japan.

*Corresponding author

E-mail: ymatsumoto@my-pharm.ac.jp (YM)

**Table S1 MIC values of *crz1* gene-deficient mutants against antifungal agents.**

| MIC (μg/mL) | Parent | *crz1 Δ*#1 | *crz1 Δ*#2 | *crz1 Δ*#3 |
|---|---|---|---|---|
| MCFG | >16 | >16 | >16 | >16 |
| CPFG | 8 | 4 | 4 | 4 |
| AMPH-B | 0.5 | 0.5 | 0.25 | 0.5 |
| 5-FC | >64 | >64 | >64 | >64 |
| FLCZ | 2 | 2 | 4 | 4 |
| ITCZ | 0.25 | 0.25 | 0.25 | 0.25 |
| VRCZ | 0.12 | 0.25 | 0.25 | 0.12 |
| MCZ | 0.5 | 0.5 | 0.5 | 0.25 |

The *T. asahii* parental strain (Parent) and *crz1* gene-deficient mutant *T. asahii* strains were cultured in Sabouraud medium for 2 days at 27 °C. *T. asahii* cells were suspended in saline, and cell suspensions were prepared to a turbidity of 1 according to the McFarland turbidity method. Each bacterial solution was diluted 20-fold in saline and then 100-fold in RPMI MOPS. The prepared fungal solutions were then diluted with eight antifungal agents (Micafungin [MCFG], caspofungin [CPFG], amphotericin B [AMPH-B], 5-fluorocytosine [5-FC], fluconazole [FLCZ], itraconazole [ITCZ], voriconazole [VRCZ], miconazole [MCZ]) and inoculated on plates at 37°C for 48 h. The minimum inhibitory concentration (MIC) was determined from the results of two independent experiments.

Descriptions | Graphic Summary | Alignments | Taxonomy

**Sequences producing significant alignments** Download Select columns Show 100

select all *11 sequences selected* GenPept Graphics Distance tree of results Multiple alignment MSA Viewer

| Description | Scientific Name | Max Score | Total Score | Query Cover | E value | Per. Ident | Acc. Len | Accession |
|---|---|---|---|---|---|---|---|---|
| hypothetical protein A1Q1_05631 [Trichosporon asahii var. asahii CBS 2479] | Trichosporon asahii var. asahii CB… | 498 | 498 | 75% | 3e-161 | 47.17% | 871 | XP_014176315.1 |
| hypothetical protein A1Q1_05726 [Trichosporon asahii var. asahii CBS 2479] | Trichosporon asahii var. asahii CB… | 72.0 | 72.0 | 10% | 4e-13 | 39.13% | 613 | XP_014176410.1 |
| hypothetical protein A1Q1_03045 [Trichosporon asahii var. asahii CBS 2479] | Trichosporon asahii var. asahii CB… | 69.3 | 69.3 | 9% | 3e-12 | 36.54% | 757 | XP_014179749.1 |
| hypothetical protein A1Q1_03458 [Trichosporon asahii var. asahii CBS 2479] | Trichosporon asahii var. asahii CB… | 54.7 | 54.7 | 14% | 1e-07 | 29.53% | 936 | XP_014178731.1 |
| hypothetical protein A1Q1_04892 [Trichosporon asahii var. asahii CBS 2479] | Trichosporon asahii var. asahii CB… | 47.4 | 47.4 | 5% | 7e-06 | 38.33% | 260 | XP_014177183.1 |
| specific RNA polymerase II transcription factor [Trichosporon asahii var. asahii CBS 2479] | Trichosporon asahii var. asahii CB… | 48.1 | 48.1 | 7% | 8e-06 | 38.75% | 417 | XP_014182549.1 |
| transcription factor iiia [Trichosporon asahii var. asahii CBS 2479] | Trichosporon asahii var. asahii CB… | 47.0 | 47.0 | 9% | 2e-05 | 33.00% | 603 | XP_014176225.1 |
| hypothetical protein A1Q1_07124 [Trichosporon asahii var. asahii CBS 2479] | Trichosporon asahii var. asahii CB… | 44.3 | 44.3 | 5% | 1e-04 | 40.35% | 736 | XP_014182848.1 |
| RNA polymerase II transcription factor [Trichosporon asahii var. asahii CBS 2479] | Trichosporon asahii var. asahii CB… | 40.8 | 40.8 | 8% | 0.001 | 29.76% | 595 | XP_014179604.1 |
| Zinc finger protein [Trichosporon asahii var. asahii CBS 2479] | Trichosporon asahii var. asahii CB… | 39.3 | 39.3 | 4% | 0.004 | 45.24% | 429 | XP_014177409.1 |
| transcriptional regulator nrg2 [Trichosporon asahii var. asahii CBS 2479] | Trichosporon asahii var. asahii CB… | 38.5 | 75.9 | 6% | 0.008 | 47.06% | 487 | XP_014182037.1 |

**Fig. S1. Estimation of Crz1 in *Trichosporon asahii* based on sequence similarity to *Cryptococcus neoformans* Crz1.**

A BLAST search (https://blast.ncbi.nlm.nih.gov/Blast.cgi) was performed against the *T. asahii* genome using the amino acid sequence of *C. neoformans* Crz1 as the query. The protein A1Q1_05631 showed a query coverage of 75%, whereas all other candidate proteins exhibited query coverage of less than 15%. The E-value for A1Q1_05631 was $e^{-161}$, while the E-values of the other proteins were greater than $e^{-13}$. Based on these results, A1Q1_05631 was estimated as Crz1 in *T. asahii*.

Descriptions | Graphic Summary | Alignments | Taxonomy

**Sequences producing significant alignments** Download Select columns Show 100

select all *99 sequences selected* GenPept Graphics Distance tree of results Multiple alignment MSA Viewer

| Description | Scientific Name | Max Score | Total Score | Query Cover | E value | Per. Ident | Acc. Len | Accession |
|---|---|---|---|---|---|---|---|---|
| hypothetical protein A1Q1_05631 [Trichosporon asahii var. asahii CBS 2479] | Trichosporon asahii var. asahii ... | 1776 | 1776 | 100% | 0.0 | 100.00% | 871 | XP_014176315.1 |
| hypothetical protein CNAG_00156 [Cryptococcus neoformans H99] | Cryptococcus neoformans H99 | 538 | 538 | 87% | 5e-176 | 48.82% | 1094 | XP_012046570.1 |
| DNA-binding transcription factor [Candida albicans SC5314] | Candida albicans SC5314 | 104 | 104 | 14% | 1e-22 | 42.11% | 731 | XP_716600.1 |
| DNA-binding transcription factor CRZ1 [Saccharomyces cerevisiae S288C] | Saccharomyces cerevisiae S288C | 101 | 101 | 11% | 6e-22 | 49.07% | 678 | NP_014371.1 |
| putative C2H2 transcription factor Crz1 [Aspergillus fumigatus Af293] | Aspergillus fumigatus Af293 | 99.0 | 99.0 | 11% | 5e-21 | 46.08% | 754 | XP_750439.1 |

**Fig. S2. Identification of Crz1 homologs in other fungal species based on the *T. asahii* A1Q1_05631 amino acid sequence.**

A BLAST search (https://blast.ncbi.nlm.nih.gov/Blast.cgi) was performed using the amino acid sequence of *T. asahii* A1Q1_05631 as the query against fungal protein databases. This analysis identified Crz1 or CrzA homologs in other fungal species, including *Cryptococcus neoformans*, *Candida albicans*, *Saccharomyces cerevisiae*, and *Aspergillus fumigatus*.

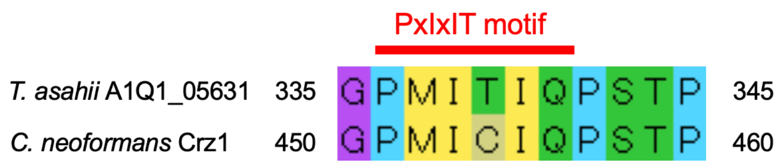


**Fig. S3. Prediction of the PxIxIT motif in *Trichosporon asahii* A1Q1_05631.**
In *Cryptococcus neoformans*, deletion of the gene region encoding residues 451–456 (PMICIQ), corresponding to the PxIxIT motif in Crz1, has been shown to abolish nuclear localization of Crz1 (1). Based on sequence analysis, residues 336–341 (PMITIQ) in *T. asahii* A1Q1_05631 were predicted to constitute the PxIxIT motif.

## Reference

1. Chow EWL, Clancey SA, Billmyre RB, Averette AF, Granek JA, Mieczkowski P, Cardenas ME, Heitman J. 2017. Elucidation of the calcineurin-Crz1 stress response transcriptional network in the human fungal pathogen *Cryptococcus neoformans*. PLOS Genetics 13:e1006667.

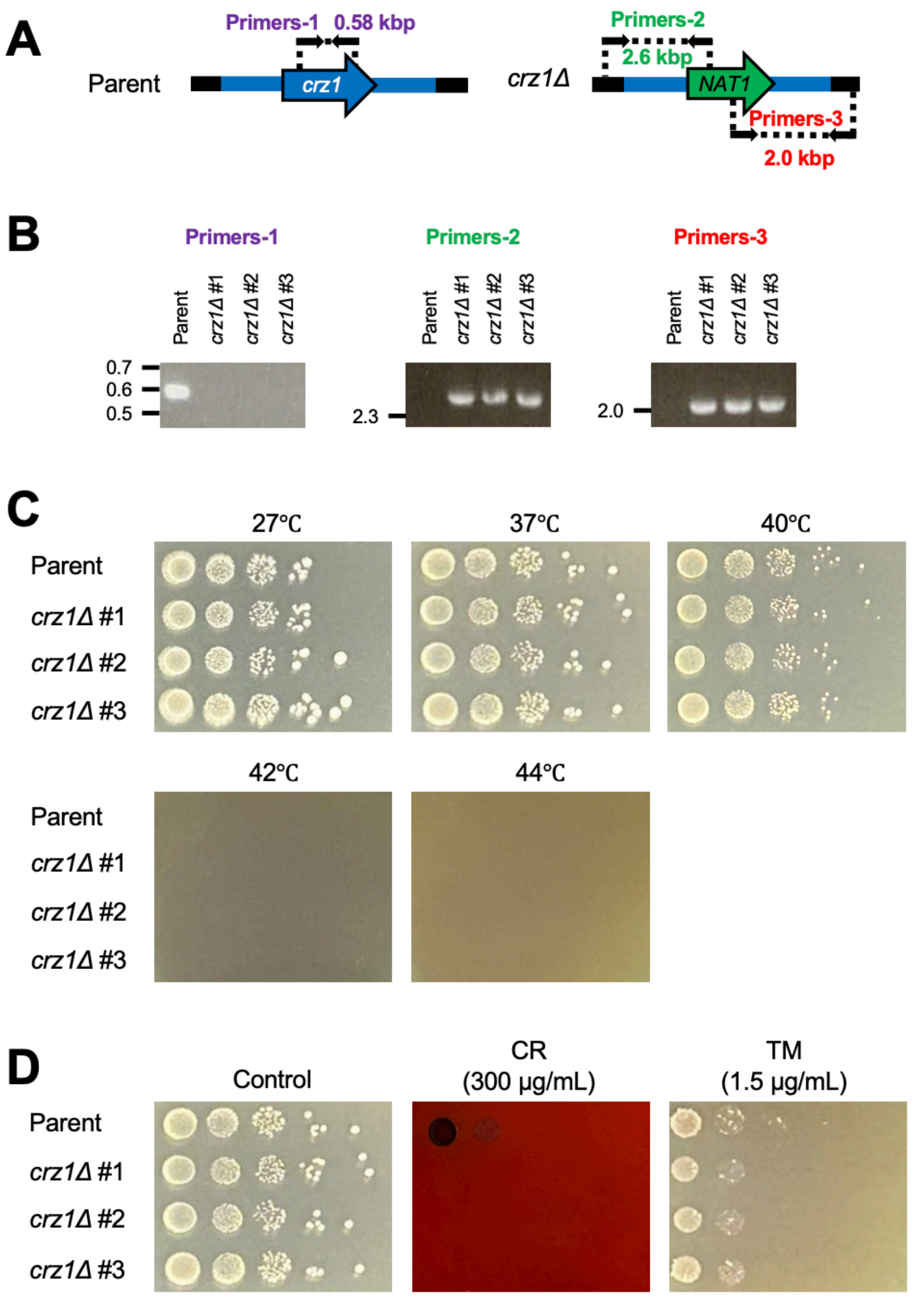


**Fig. S4. Stress sensitivity analysis of three independent *crz1* gene-deficient mutants in *Trichosporon asahii*.**

(A) Schematic representation of primer positions used for PCR verification of the genomic structure of the *crz1* gene-deficient mutant and the expected sizes of the amplified DNA fragments. (B) Agarose gel electrophoresis of PCR products confirming the genotypes of the *crz1* gene-deficient mutants. (C) The parental strain (Parent) and three independent *crz1* gene-deficient mutants (*crz1*Δ #1–3) were cultured on Sabouraud dextrose agar (SDA) at 27°C for 2 days. Cells were suspended in physiological saline,

and serial 10-fold dilutions were prepared. Aliquots (5 µL) of each dilution were spotted onto SDA plates and incubated at 27°C, 37°C, and 40°C for 72 h, or at 42°C and 44°C for 120 h. Neither the parental strain (Parent) nor the *crz1* gene-deficient mutants (*crz1*Δ #1–3) grew at 42°C or 44°C. Therefore, stress sensitivity at these temperatures could not be evaluated for the *crz1* gene-deficient mutants at 42°C and 44°C. (D) The parental strain (Parent) and the three *crz1* gene-deficient mutants (*crz1*Δ #1–3) were cultured as described above, serially diluted, and spotted onto SDA containing tunicamycin (TM) or Congo red (CR). Plates were incubated at 37°C for 72 h. All three *crz1* gene-deficient mutants consistently exhibited increased sensitivity to TM and CR.
